# A novel delta current method for transport stoichiometry estimation

**DOI:** 10.1186/s13628-014-0014-2

**Published:** 2014-12-11

**Authors:** Xuesi M Shao, Liyo Kao, Ira Kurtz

**Affiliations:** Division of Nephrology, Department of Medicine, David Geffen School of Medicine at UCLA, Los Angeles, CA 90095 USA; Department of Neurobiology, David Geffen School of Medicine at UCLA, Los Angeles, CA 90095 USA; Brain Research Institute, David Geffen School of Medicine at UCLA, Los Angeles, CA 90095 USA

**Keywords:** Electrogenic transporter, Stoichiometry, Membrane current-voltage relationship, Reversal potential, HEK-293 cells, Patch clamp, Computational simulation

## Abstract

**Background:**

The ion transport stoichiometry (q) of electrogenic transporters is an important determinant of their function. q can be determined by the reversal potential (E_rev_) if the transporter under study is the only electrogenic transport mechanism or a specific inhibitor is available. An alternative approach is to calculate delta reversal potential (ΔE_rev_) by altering the concentrations of the transported substrates. This approach is based on the hypothesis that the contributions of other channels and transporters on the membrane to E_rev_ are additive. However, E_rev_ is a complicated function of the sum of different conductances rather than being additive.

**Results:**

We propose a new delta current (ΔI) method based on a simplified model for electrogenic secondary active transport by Heinz (*Electrical Potentials in Biological Membrane Transport*, 1981). ΔI is the difference between two currents obtained from altering the external concentration of a transported substrate thereby eliminating other currents without the need for a specific inhibitor. q is determined by the ratio of ΔI at two different membrane voltages (V_1_ and V_2_) where q = 2RT/(F(V_2_ –V_1_))ln(ΔI_2_/ΔI_1_) + 1. We tested this ΔI methodology in HEK-293 cells expressing the elctrogenic SLC4 sodium bicarbonate cotransporters NBCe2-C and NBCe1-A, the results were consistent with those obtained with the E_rev_ inhibitor method. Furthermore, using computational simulations, we compared the estimates of q with the ΔE_rev_ and ΔI methods. The results showed that the ΔE_rev_ method introduces significant error when other channels or electrogenic transporters are present on the membrane and that the ΔI equation accurately calculates the stoichiometric ratio.

**Conclusions:**

We developed a ΔI method for estimating transport stoichiometry of electrogenic transporters based on the Heinz model. This model reduces to the conventional reversal potential method when the transporter under study is the only electrogenic transport process in the membrane. When there are other electrogenic transport pathways, ΔI method eliminates their contribution in estimating q. Computational simulations demonstrated that the ΔE_rev_ method introduces significant error when other channels or electrogenic transporters are present and that the ΔI equation accurately calculates the stoichiometric ratio. This new ΔI method can be readily extended to the analysis of other electrogenic transporters in other tissues.

## Background

Based on their electrical properties, membrane protein transporters are classified as being either electrogenic (transport a net charge) or electroneutral [1-3]. Which of these categories a given transporter belongs to is dependent on its substrate (or ion) coupling ratio; its transport stoichiometry represented by the symbol q. Electrogenic transporters are sensitive to both the electrical and chemical gradients of the ions that are being transported across a membrane. Unlike electroneutral transporters, electrogenic transporters can utilize the membrane potential of a cell or organelle membrane to drive substrates or ions against their chemical gradients. For a given electrochemical gradient, the transport stoichiometry is therefore an important independent determinant of both the magnitude and direction of substrate or ion flux through a membrane transport protein. The simplest stoichiometry for an electrogenic transporter is 1:1 as in the case of the sodium-coupled glucose transporter SGLT2 [4]. In many instances more complex stoichiometries have been reported [4,5]. Furthermore, certain transporters have variable stoichiometry ratios [6-10].

The most intuitively straightforward approach for measuring the stoichiometry of a transporter is to measure the flux of each transported species either directly [11] or indirectly [12]. In many instances, technical difficulties or sensitivity/specificity considerations preclude interpretable flux measurements from being acquired. Rather than measuring the actual substrate fluxes, a widely used approach is to measure the steady state current-voltage (I-V) properties of the transporter. In this approach, one determines the reversal potential (E_rev_), and estimates q as for example in the case of an electrogenic sodium coupled bicarbonate transporter [1] as follows:

1 $$ {E}_{NBC}=\frac{RT}{F\left(q-1\right)} \ln \frac{{\left[N{a}^{+}\right]}_i{\left({\left[HC{O_3}^{-}\right]}_i\right)}^q}{{\left[N{a}^{+}\right]}_o{\left({\left[HC{O_3}^{-}\right]}_o\right)}^q} $$

where intracellular concentrations of Na^+^ ([Na^+^]_i_) and HCO_3_^−^ ([HCO_3_^−^]_i_) as well as extracellular concentrations of Na^+^ ([Na^+^]_o_) and HCO_3_^−^ ([HCO_3_^−^]_o_) are known and E_NBC_ is the reversal potential of the transporter. F, R and T are Faraday’s constant, gas constant and absolute temperature respectively. RT/F = 25.69 at 25°C [13].

If the electrogenic transporter under consideration is the only transport mechanism in the membrane, q estimated by solving Eq. 1 is accurate. In most cells or expression systems, there are other channels or electrogenic transporters in the membrane, reversal potential method requires the use of a specific inhibitor to differentiate the transport process of interest from other transport pathways. Subtracting the I-V curve in the presence of the inhibitor from the I-V curve without inhibitor, one obtains the E_rev_ of the transporter-mediated current. Therefore, the relationship of Eq. 1 still holds.

Given that inhibitors are not always as specific as one would prefer, or in circumstances where a specific inhibitor is unavailable, an alternative approach has been to measure the change in zero-current membrane potential (V_I=0_, the voltage of the I-V curve measured at I = 0), by altering the chemical gradient(s) of the transported species [15-16]. Then ΔE_rev_ is

$$ \varDelta {E}_{rev}={V}_{I=0}\kern0.5em  at\ a\  concentration\  of\ a\  substrate-{V}_{I=0}\kern0.5em  at\kern0.5em  another\kern0.5em  concentration $$

There are some variations of the ΔE_rev_ approach such as estimating q by determining the slope of V_I=0_ vs. ion or substrate concentrations [2]. In this report, we show that ΔE_rev_ approach is correct only when the transport current under study is the only current in the membrane or in other words, currents mediated by other channels, electrogenic transporters, and leak current are negligible. When the currents mediated by other channels/transporters are not negligible, the implicit assumption underlying the ΔE_rev_ approach and its variations is that the reversal potentials due to other channels and transporters are additive to the E_rev_ of the transporter under study, therefore they can be eliminated by subtraction. However, the assumption that E_rev_ is additive is not valid since the effect of multiple channels/electrogenic transporters on ΔE_rev_ is a complicated function of the concentrations of ions and substrates involved, as well as the conductance and transport rate of those pathways [17,18].

To address these issues, we have developed a new approach named the “delta current (ΔI) method”. The utility of the ΔI approach is demonstrated using the electrogenic sodium bicarbonate cotransporters NBCe2-C and NBCe1-A [14,19-21] expressed in HEK-293 cells. In vivo, NBCe2-C is expressed in choroid plexus epithelial cells and other tissues. NBCe1-A is expressed in the mammalian kidney proximal tubule and the eye. This method has several advantages: 1) The equation does not suffer from the aforementioned errors in the ΔE_rev_ method due to other channels and functional electrogenic transporters; 2) Like the ΔE_rev_ method, the measurement protocol does not require a specific inhibitor. In addition, by computational simulations, we show the advantage of the ΔI method in calculating the stoichiometry ratio of an electrogenic transporter, and demonstrate that the ΔE_rev_ method can introduce significant errors in estimating q.

## Methods

### Expression of NBCe2-C and NBCe1-A in HEK-293 cells

The SLC4 human NBCe2-C and NBCe1-A proteins were expressed in HEK-293 cells as follows. Full-length human cDNA for each transporter was cloned into a pMSCV-IRES-EGFP (Clontech, Mountain View, CA) which expresses the transporters under a CMV promoter and also expresses EGFP as a separate protein under an internal ribosome entry site. The cDNA sequence of each of the constructs was verified by DNA sequencing. Use of human material and cell line are approved by UCLA Institutional Biosafety Committee (IBC#111.13.0-r).

### Electrophysiological recordings

Cells expressing each transporter were cultured in DMEM media with 5% FBS/5% CO_2_ and 37°C. The cells were transferred to 35 mm tissue culture (Bioptechs, Butler PA) inserts that were placed on the microscope stage for patch-clamp recording. The cells were continually superfused with bath solution (~2 ml/min) during the experiments. All experiments were performed in room temperature (22 ± 1°C). HEK-293 cells were whole-cell patch-clamped with the aid of fluorescent optics (Axioskop2, Carl Zeiss, Göttingen, Germany). Patch pipettes were pulled from thick wall (0.32 mm) borosilicate glass with tip size 1 - 1.5 μm (resistance: 4-6.5 MΩ). The patch pipette filling solution and bath solution components are listed in Table 1. All solutions were pH 7.4 that were confirmed with pH meter measurements in conditions throughout the studies. To ensure stable electrode potentials during whole-cell patch-clamp recordings, a micro-agar salt bridge of 2 M KCl was built in the electrode holder that formed an electrical connection between the pipette solution and the Ag/AgCl wire connected to the headstage of a patch-clamp amplifier [22]. Intracellular signals were amplified and low pass-filtered at 400 Hz with a patch-clamp amplifier (MultiClamp 700B, Molecular Devices Co., Sunnyvale, CA). Whole cell capacitance and series resistance were determined with the auto whole-cell capacitance and series resistance compensation. The series resistance was usually compensated 80% (both prediction and correction). Junction potentials generated by different pairs of patch pipette solutions and bath solutions were determined with the junction potential calculator in software Clampex 10 (Molecular Devices Co., Sunnyvale, CA) and reported potential values were corrected for junction potentials. The inhibitor 4,4′-Diisothiocyanatostilbene-2,2′-disulfonic acid disodium salt (DIDS; SIGMA-Aldrich Co., St. Louis, MO.) was used to block NBCe2-C and NBCe1-A function.

**Table 1:** **Solutions**

**Components**	**Pipette**				**Bath**				
	**a**	**b**	**c**	**d**	**A**	**B**	**C**	**D**	**E**
NaCl					110	110	55		15
CsCl					10	10	10		
CaCl_2_	1	1	1	1	1.5	1.5	1.5	1.5	1.5
MgCl_2_					1	1	1	1	1
TEA-Cl	10	10	10	10					
TMA-Cl							55	120	105
EGTA	10	10	10	10					
HEPES	10	50	50	50	10	10	10	10	10
NaHCO_3_		8	25	25		25	25	10	10
Cs-Gluconate	125	105	105	90					
Cs-HCO_3_		17							
TMA-HCO_3_								15	15
Na-Gluconate	10			15	25				
ATP-Mg	1	1	1	1					
ATP-Na_2_	1	1							
Glucose					15	15	15	15	15

Bicarbonate-containing solutions were bubbled with 5% CO_2_ and 95% O_2_. All solutions were pH 7.4. Glucose was included in the bath solutions to adjust the osmolality to approximately 300 mmol/Kg. The solution osmolality was determined with an osmometer (Model 5520, Vapro® vapor pressure osmometer, Wescor Inc., Logan, UT, USA).

### Data analysis

Signals from intracellular recordings were digitized at 2 KHz sampling frequency with the Digidata 1440A and software Clampex 10 (Molecular Devices Co., CA, USA). The signals were saved as data files for further analyses off-line. Data are expressed as mean ± SE. Paired t-test was used for determining statistical significance. p ≤ 0.05 was taken as the criterion for significance.

## Results

### Estimation of NBCe2-C transport stoichiometry with the conventional reversal potential method

**Figure 1:**
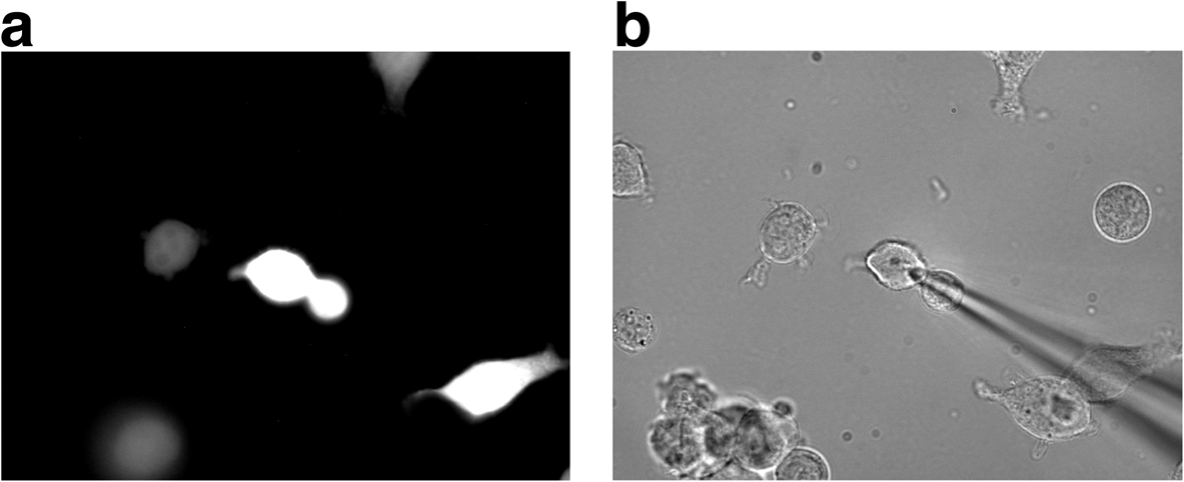
**The microscope image of cultured HEK-293 cells and corresponding fluorescent image of the same field. a)** Fluorescent microscopic image of HEK-293 cells expressing NBCe2-C and EGFP under separate promoters. **b)** Light microscope image showing the electrode patched on an EGFP positive cell.

**Figure 2:**
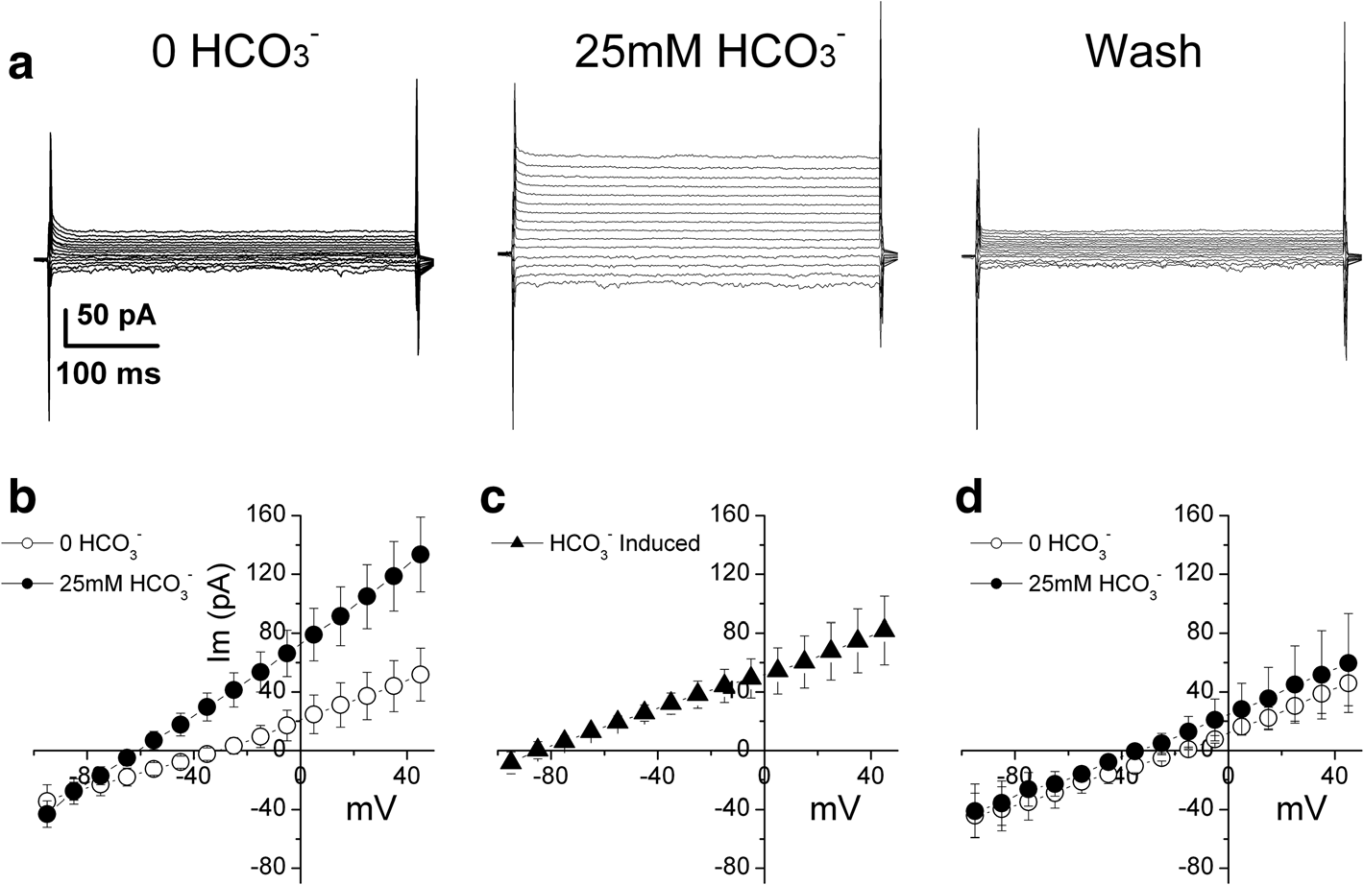
**HCO**_**3**_^**−**^ **-induced current in NBCe2-C expressing HEK-293 cells. a)** The cell was whole-cell voltage-clamped at -60 mV. A series of 400 ms voltage-clamp pulses range from -95 to +45 mV with increment of 10 mV were applied and whole-cell current responses were recorded. In the pre-HCO_3_^−^ conditions, there is no HCO_3_^−^ in the patch pipette (Table 1, patch solution a) nor in the bath solution (Table 1, Bath solution A). Increasing HCO_3_^−^ concentration to 25 mM in the bath solution (Bath solution B in Table 1) induced a voltage-dependent current (central panel). The current recovered when the cell was washed with solution containing 0 HCO_3_^−^ (right panel). **b)** Current-voltage (I-V) relation of steady-state current in the absence and presence of HCO_3_^−^ (n = 8). Im (pA): membrane current in pA. Steady-state current was obtained by averaging 80 ms of the current trace toward the end of each 400 ms voltage pulse. **c)** I-V curve of HCO_3_^−^ induced current is the difference between the I-V curves in the absence of HCO_3_^−^ and in the presence of HCO_3_^−^. **d)** Application of 25 mM HCO_3_^−^ in the bath did not induce any current in EGFP negative cells (n = 4).

The light microscopic image of cultured HEK-293 cells and corresponding fluorescent image of the same field is shown in Figure 1a and b respectively. Bright fluorescent cells were EGFP positive and thus were NBCe2-C expressing cells as well. We voltage-clamped EGFP positive cells at a holding voltage -60 mV and applied a series of 400 ms pulses from -95 to +45 with increment of 10 mV. The current responses to the series of pulses in pre-HCO_3_^−^ (0 HCO_3_^−^) conditions were background current due to endogenous channels in HEK-293 cells (Figure 2a left panel). We established an I-V curve of steady state current. Figure 2b shows the mean I-V curves from 8 cells. The steady state current at +45 mV was 51.8 ± 18.0 pA (mean ± SE, n = 8). Bath application of a solution containing 25 mM HCO_3_^−^ (Table 1, bath solution B) induced a voltage-dependent current (Figure 2a central panel). The mean I-V curve in the presence of HCO_3_^−^ is shown in Figure 2b. The steady state current at voltage +45 mV was 133.5 ± 25.5 pA (p = 0.01, paired t-test vs pre-HCO_3_^−^). The HCO_3_^−^-induced current was obtained by subtracting the current traces in the absence of HCO_3_^−^ from the current traces in its presence. Figure 2c shows the mean I-V curve of HCO_3_^−^ induced current. The mean HCO_3_^−^-induced current at voltage +45 mV was 81.7 ± 23.3 pA (n = 8). The current was greatly reduced after washing with the control bath solution (Figure 2a right panel). As a separate control, we tested whether the application of HCO_3_^−^ containing solution induced any current in EGFP negative cells. As shown in Figure 2d, there is no significant HCO_3_^−^-induced current detected in these cells (n = 4). These results indicate that functional NBCe2-C is expressed in EGFP labeled HEK-293 cells and that NBCe2-C transports HCO_3_^−^ electrogenically.

To estimate the NBCe2-C HCO_3_^−^ to Na^+^ transport stoichiometry q, the conventional method of measuring the reversal potential with the inhibitor DIDS was used initially. At known intracellular and extracellular concentrations of Na^+^ and HCO_3_^−^, q could be estimated with Eq. 1.

**Figure 3:**
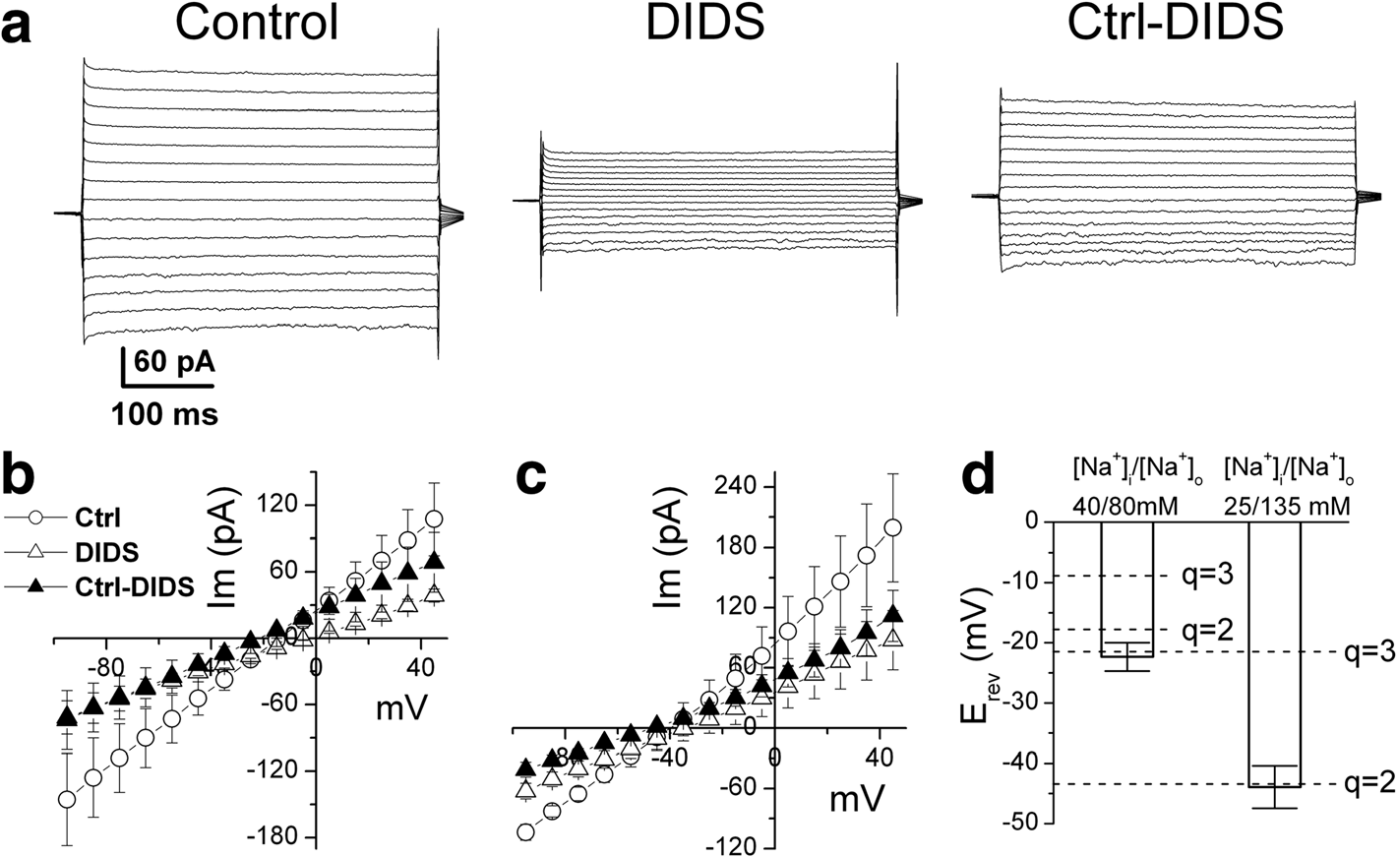
**Estimation of transport stoichiometry for NBCe2-C using conventional reversal potential method. a)** In the conditions of equal concentrations (25 mM) of HCO_3_^−^ intra- and extracellularly, the ratio of intracellular concentration of Na^+^ ([Na^+^]_i_) and extracellular concentration of Na^+^ ([Na^+^]_o_) = 40/80 mM (Patch solution d/bath solution C in Table 1), cells were voltage-clamped at -60 mV. Current responses to a series of 400 mV voltage pulses from -95 to +45 mV with increment of 10 mV were recorded in the absence (pre-DIDS Control, Ctrl) and presence of DIDS (0.5 mM). DIDS sensitive current (right panel, Ctrl-DIDS) was obtained by digital subtraction of currents in the presence of DIDS (center panel) from control current (n = 3). B) I-V relations of steady-state current in Ctrl, DIDS and Ctrl-DIDS conditions. **c)** I-V curves obtained with the same protocol as **(b)** except [Na^+^]_i_/[Na^+^]_o_ = 25/135 mM (Patch solution c/bath solution B in Table 1) (n = 5). **d)** The two V_I=0_ values are close to the calculated values assuming q = 2 (dash lines) while significantly distinct from the calculated values assuming q = 3 (dash lines).

In this study, HEK-293 cells expressing NBCe2-C were whole-cell patch-clamped at -60 mV. V_I=0_ was measured in two independent experiments where [HCO_3_^−^]_i_ and [HCO_3_^−^]_o_ were equal (25 mM), therefore E_NBC_ depended only on [Na^+^]_i_/[Na^+^]_o_. For every cell recorded, we waited at least 10 min from establishment of whole-cell patch-clamp to ensure that [Na^+^]_i_ and [HCO_3_^−^]_i_ were equal to the concentrations of Na^+^ and HCO_3_^−^ respectively in the patch pipette solution by diffusion before beginning I-V measurement. Current responses to a series of voltage pulses were recorded to establish I-V relationship in the absence and presence of DIDS (0.5 mM, Figure 3a). In the first experiment, using [Na^+^]_i_/[Na^+^]_o_ = 40/80 mM (Patch solution d/bath solution C in Table 1), I-V curve of steady-state NBCe2-C transport current (DIDS sensitive current) was obtained by subtraction of currents in the presence of DIDS from control current (pre-DIDS). V_I=0_ = -22.3 ± 2.4 mV (n = 3) was obtained (Figure 3a,b and d). To show the mean and variability among cells, this V_I=0_ value was averaged from the V_I=0_ of individual sample cells. Note that this mean V_I=0_ value is very close to the V_I=0_ points where the average DIDS-sensitive I-V curve crosses the x-axis in (Figure 3b). In the second experiment using [Na^+^]_i_/[Na^+^]_o_ = 25/135 mM (Patch solution c/bath solution B in Table 1), we got V_I=0_ = -43.9 ± 3.5 mV (n = 5, Figure 3c and d). The two V_I=0_ values are close to the calculated E_NBC_ values of -17.8 and -43.3 mV (Eq. 1), respectively, assuming q = 2 (dash lines) while significantly distinct from the calculated values assuming q = 3 (dash lines, Figure 3d). The results indicate that the transport stoichiometry ratio of NBCe2-C is 2 HCO_3_^−^: 1 Na^+^ or (1 CO_3_^2−^: 1 Na^+^) in HEK-293 cells.

### A novel delta current method for estimation of transport stoichiometry

Based on a simplified model for electrogenic secondary active transport [23] (as originally applied to the Na^+^/Ca^2+^ transporter), in the case of an electrogenic NBC transporter, the Na^+^-HCO_3_^−^ flux (J_c_) is shown in Eq. 2. Although we limit our evidence for the validity of our method to electrogenic NBC transporters, the approach is applicable to other electrogenic transporters.

2 $$ \begin{array}{l}{J}_c={K}_c\left\{{\left({\left[N{a}^{+}\right]}_o \exp \left(-\frac{FV}{RT}\frac{z_{Na}}{2}\right)\right)}^{\nu_{Na}}\times {\left({\left[HC{O_3}^{-}\right]}_o \exp \left(-\frac{FV}{RT}\frac{z_{HC{O}_3}}{2}\right)\right)}^{\nu_{HC{O}_3}}\right.\\ {}\left.-{\left({\left[N{a}^{+}\right]}_i \exp \left(-\frac{FV}{RT}\frac{-{z}_{Na}}{2}\right)\right)}^{\nu_{Na}}\times {\left({\left[HC{O_3}^{-}\right]}_i \exp \left(-\frac{FV}{RT}\frac{-{z}_{HC{O}_3}}{2}\right)\right)}^{\nu_{HC{O}_3}}\right\}\end{array} $$

where K_c_ is an involved function of mobility and concentrations of free and loaded carrier [23] (also refer to [24]). z_Na_ is the valence of Na^+^ and ν_Na_ is the stoichiometry of Na^+^. ν_HCO3_ is the stoichiometry of HCO_3_^−^. V is the membrane potential. The total membrane current is:

3 $$ \begin{array}{l}{I}_M=F{K}_c\left\{{\left({\left[N{a}^{+}\right]}_o \exp \left(-\frac{FV}{2RT}\right)\right)}^{\nu_{Na}}\times {\left({\left[HC{O_3}^{-}\right]}_o \exp \left(\frac{FV}{2RT}\right)\right)}^{\nu_{HC{O}_3}}\right.\\ {}\left.-{\left({\left[N{a}^{+}\right]}_i \exp \left(\frac{FV}{2RT}\right)\right)}^{\nu_{Na}}\times {\left({\left[HC{O_3}^{-}\right]}_i \exp \left(-\frac{FV}{2RT}\right)\right)}^{\nu_{HC{O}_3}}\right\}+{\displaystyle \sum_j{I}_j}\end{array} $$

Where ${\displaystyle \sum_j{I}_j}$ is the sum of all other currents mediated by various channels and electrogenic transporters including leak current on the membrane. ${\displaystyle \sum_j{I}_j}$ can be a non-linear function of V while a general assumption is that it is independent of NBC transport current.

If we change the Na^+^ concentration outside the cell from [Na^+^]_o1_ to [Na^+^]_o2_, the whole cell current would change from I_M1_ to I_M2_. We assume that K_c_ does not vary with [Na^+^]_o_ within a range far from saturation. We also assume that the sum of other currents ${\displaystyle \sum_j{I}_j}$ is a function of V while the function is unchanged when [Na^+^]_o_ changes (see Discussion). Therefore the delta current is

4 $$ \begin{array}{l}\varDelta {I}_M={I}_{M2}-{I}_{M1}=F{K}_c\left\{{\left({\left[N{a}^{+}\right]}_{o2} \exp \left(-\frac{FV}{2RT}\right)\right)}^{\nu_{Na}}\times {\left({\left[HC{O_3}^{-}\right]}_o \exp \left(\frac{FV}{2RT}\right)\right)}^{\nu_{HC{O}_3}}\right.\\ {}\left.-{\left({\left[N{a}^{+}\right]}_{o1} \exp \left(-\frac{FV}{2RT}\right)\right)}^{\nu_{Na}}\times {\left({\left[HC{O_3}^{-}\right]}_o \exp \left(\frac{FV}{2RT}\right)\right)}^{\nu_{HC{O}_3}}\right\}\end{array} $$

${\displaystyle \sum_j{I}_j}$ is completely eliminated. For simplicity, we take ν_Na_ = 1 and q = ν_HCO3_/ν_Na_.

Now we consider at two different voltage points V_1_ and V_2_, we have two ΔI_M_ values, ΔI_V1_ and ΔI_V2_. We take the ratio of them,

5 $$ \frac{\varDelta {I}_{V2}}{\varDelta {I}_{V1}}=\frac{F{K}_c\left\{\left({\left[N{a}^{+}\right]}_{o2}-{\left[N{a}^{+}\right]}_{o1}\right) \exp \left(-\frac{F{V}_2}{2RT}\right)\cdot {\left({\left[HC{O_3}^{-}\right]}_o \exp \left(\frac{F{V}_2}{2RT}\right)\right)}^q\right\}}{F{K}_c\left\{\left({\left[N{a}^{+}\right]}_{o2}-{\left[N{a}^{+}\right]}_{o1}\right) \exp \left(-\frac{F{V}_1}{2RT}\right)\cdot {\left({\left[HC{O_3}^{-}\right]}_o \exp \left(\frac{F{V}_1}{2RT}\right)\right)}^q\right\}} $$

ΔI_V1_ and ΔI_V2_ can be measured in electrophysiological experiments, therefore, there is only one unknown q. q can be expressed as

6 $$ q=\frac{2RT}{F\left({V}_2-{V}_1\right)} \ln \frac{\varDelta {I}_{V2}}{\varDelta {I}_{V1}}+1 $$

In practical situations, to minimize the effect of the possible voltage dependence of K_c_ on the measurement of ΔI_M_ and estimation of q, we take [Na^+^]_o1_ = [Na^+^]_i_ and [HCO_3_^−^]_o_ = [HCO_3_^−^]_i_, where

$$ {I}_M={\displaystyle \sum_j{I}_j\kern0.5em  at\kern0.5em V=0.} $$

Therefore, at V = 0, the delta current ΔI_V1=0_ is the pure NBC transport current at [Na^+^]_o2_.

q is as simple as

7 $$ q=\frac{2RT}{F{V}_2} \ln \frac{\varDelta {I}_{V2}}{\varDelta {I}_{V1=0}}+1 $$

In the following applications, to minimize the effects of possible K_c_ voltage dependence, we also take a V_2_ value close to 0 (e.g. ± 10 to 15 mV). Therefore the calculation involves only experimental measurements of currents close to equilibrium conditions.

### Transport stoichiometry of NBCe2-C estimated with the delta current method

**Figure 4:**
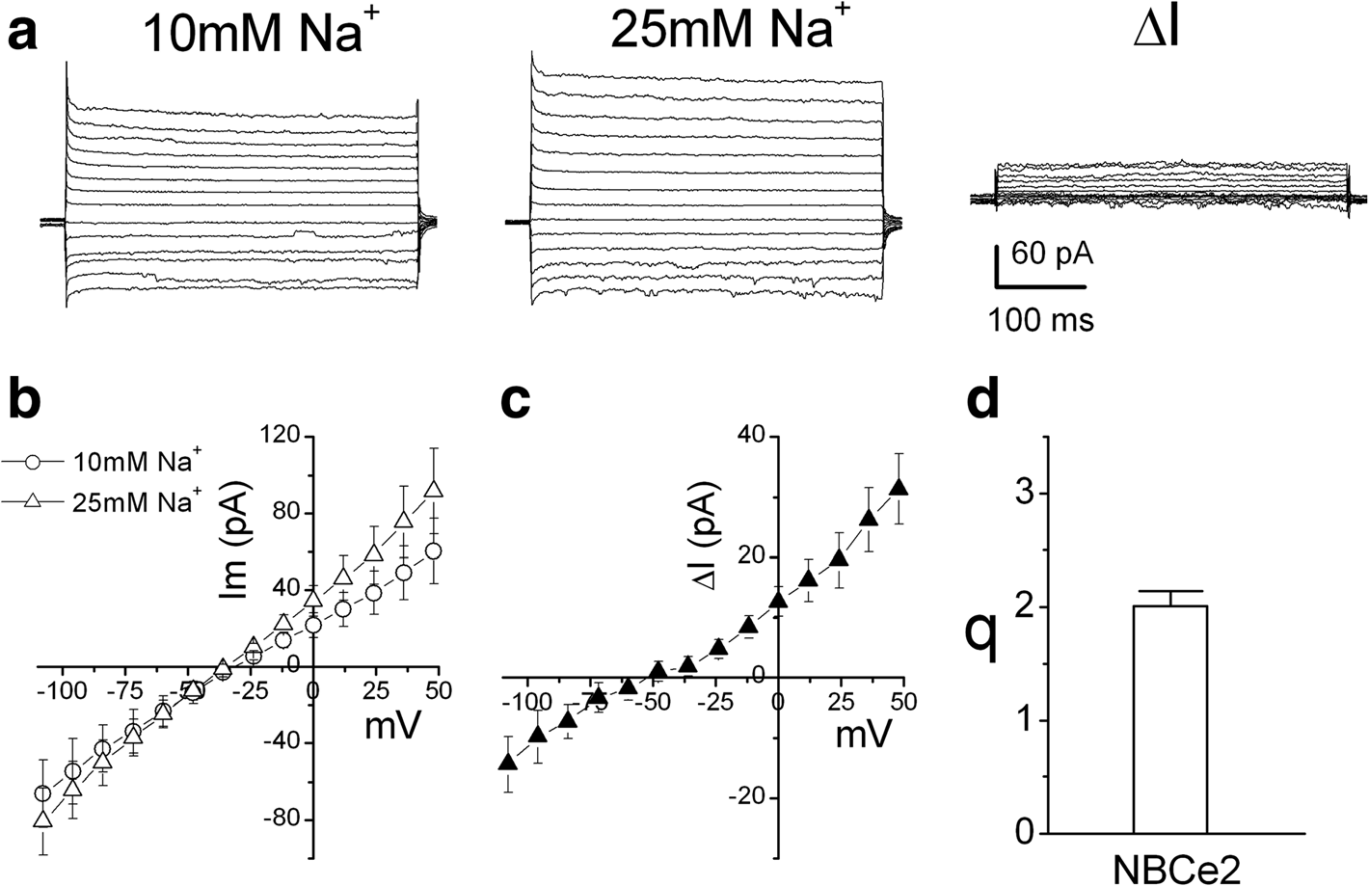
**Estimation of transport stoichiometry for NBCe2-C using the delta current method. a)** NBCe2-C expressing cells were voltage clamped at -50 mV. A series of 400 ms voltage-clamp pulses range from -108 to +48 mV with increment of 12 mV (containing a pulse to 0 mV during this protocol) was applied and whole-cell current responses were recorded. Patch pipette solution contained 10 mM Na^+^ and 25 mM HCO_3_^−^ (Solution b in Table 1). Bath solution also contained 10 mM Na^+^ and 25 mM HCO_3_^−^ (Bath solution D in Table 1) (left panel). Enhancing Na^+^ concentration from 10 to 25 mM in the bath solution (Bath solution E in Table 1) increased voltage-dependent current (central panel). Net current (ΔI) through NBCe2-C induced by changing [Na^+^]_o_ is obtained by subtracting the current traces at [Na^+^]_o_ = 10 mM from the current traces at [Na^+^]_o_ = 25 mM (right panel). **b)** Current-voltage (I-V) relations of steady-state current (mean of 80 ms current trace toward the end of each voltage pulse) in bath solutions containing 10 mM and 25 mM Na^+^. **c)** I-V relation of ΔI. **d)** Estimation of transport stoichiometry ratio q with Eq. 7 (n = 5).

Under the conditions that [Na^+^]_i_ = [Na^+^]_o_ = 10 mM and [HCO_3_^−^]_i_ = [HCO_3_^−^]_o_ = 25 mM (patch solution b and bath solution D in Table 1), NBCe2-C expressing HEK-293 cells were voltage-clamped at -50 mV and a series of voltage (including a pulse to 0 mV) was applied (Figure 4a, left panel). Increasing the Na^+^ concentration from 10 to 25 mM in the bath solution (bath solution E in Table 1) increased the voltage-dependent current (Figure 4a, central panel). Net current (ΔI) through NBCe2-C induced by changing [Na^+^]_o_ was obtained by subtracting the currents in bath solution containing 10 mM Na^+^ from currents in 25 mM [Na^+^]_o_ (Figure 4a, right panel). With this operation, according to Eq. 4, currents mediated by other channels and electrogenic transporters were eliminated if the two assumptions associated with Eq. 4 were satisfied. Figure 4b shows current-voltage (I-V) relation of steady-state current in bath solution containing 10 mM or 25 mM [Na^+^]_o_ and Figure 4c shows ΔI of NBCe2-C vs. voltages. Taking ΔI_v1_ at V = 0 and ΔI_v2_ at V = 12 mV, q is calculated using Eq. 7. We obtained q = 2.0 ± 0.14 (n = 5, Figure 4d). The results suggest that the transport stoichiometry ratio of NBCe2-C is 2 HCO_3_^−^: 1 Na^+^ (or 1 CO_3_^2−^: 1 Na^+^) in HEK-293 cells. This result is consistent with the q value obtained with the conventional reversal potential method using the inhibitor DIDS (Figure 3).

### Transport stoichiometry of NBCe1-A estimated with the delta current method

**Figure 5:**
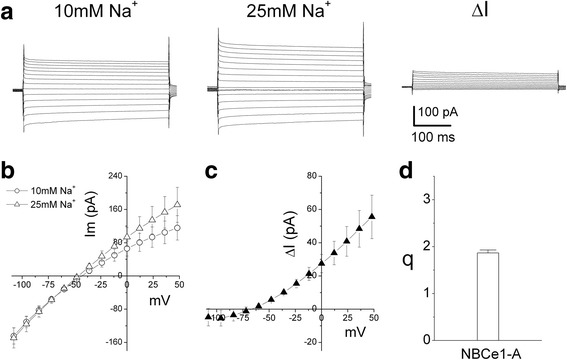
**Estimation of transport stoichiometry for NBCe1-A using the delta current method. a)** NBCe1-A expressing cells were voltage clamped at -50 mV. A series of 400 ms voltage-clamp pulses range from -108 to +48 mV with increment of 12 mV (containing a pulse to 0 mV during this protocol) was applied and whole-cell current responses were recorded. Patch pipette solution contained 10 mM Na^+^ and 25 mM HCO_3_^−^ (Solution b in Table 1). Bath solution also contained 10 mM Na^+^ and 25 mM HCO_3_^−^ (Bath solution D in Table 1) (left panel). Enhancing Na^+^ concentration from 10 to 25 mM in the bath solution (Bath solution E in Table 1). increased voltage-dependent current (central panel). Net current (ΔI) through NBCe1-A induced by changing [Na^+^]_o_ was obtained by subtracting the current traces at [Na^+^]_o_ = 10 mM from the current traces at [Na^+^]_o_ = 25 mM (right panel). **b)** Current-voltage (I-V) relations of steady-state current (mean of 80 ms current trace toward the end of each voltage pulse) in bath solutions containing 10 mM and 25 mM Na^+^. **c)** I-V relation of ΔI. **d)** Estimation of transport stoichiometry ratio q with Eq. 7 (n = 6).

Cells expressing NBCe1-A were voltage-clamped at -50 mV, and whole-cell currents were recorded when a series of voltage pulses was applied (Figure 5a). Using the same conditions as above that [Na^+^]_i_ = [Na^+^]_o_ = 10 mM and [HCO_3_^−^]_i_ = [HCO_3_^−^]_o_ = 25 mM (patch solution b and bath solution D in Table 1), increasing the Na^+^ concentration from 10 to 25 mM in the bath solution (bath solution was switched from solution D to solution E of Table 1) increased voltage-dependent current (Figure 5a middle panel). The net current (ΔI) through NBCe1-A induced by changing [Na^+^]_o_ (right panel of Figure 5a) was obtained by subtracting the current traces in the solution containing 10 mM [Na^+^]_o_ from those in 25 mM [Na^+^]_o_. The current-voltage (I-V) relation of steady-state currents in bath solution containing 10 mM or 25 mM Na^+^ is shown in Figure 5b). Figure 5c shows ΔI of NBCe1-A vs. membrane voltages. This was the result of operation of Eq. 4 and the currents mediated by other channels and electrogenic transporters were eliminated. Taking ΔI_V1_ at V = 0 and ΔI_V2_ at V = 12 mV, we calculated q using Eq. 7 for every cell. We determined q = 1.87 ± 0.062 (n = 6, Figure 5d). The results indicate that the transport stoichiometry ratio of NBCe1-A is 2 HCO_3_^−^: 1 Na^+^ or 1 CO_3_^2−^: 1 Na^+^ in HEK-293 cells. This estimate is consistent with our previous results using the conventional reversal potential method with DIDS [25].

### Computational simulation: ΔI method estimates q accurately when there are additional conductances other than electrogenic NBC transport

In native tissue or expression systems such as oocytes or HEK-293 cells, there are endogenous channels and electrogenic transporters other than the one under study. In these cases, the Δ current method is based on the assumption of additivity of membrane currents while the ΔE_rev_ method and its variations based on the assumption of additivity of reversal potentials [2,15,16]. Were the latter true, by altering the concentrations of the transported species, the contribution of other channels and electrogenic transporters could be subtracted and the relationship between delta E_rev_ and transported species concentrations and the transport stoichiometry easily obtained based on Eq. 1. This method, although widely used, is not consistent with Goldman-Hodgkin-Katz (GHK) theory [17,18] where E_rev_ is a logarithmic function of sum of concentrations of ions inside and outside of the membrane; i.e. not additive.

Now, suppose there is one kind of channel that is permeable to a univalent ion with valence z_s_ and permeability of P_s_ on the cell membrane, in addition to an electrogenic NBC transporter. Based on Eq. 2 and the GHK current equation (with all original GHK assumptions applied [18]), the current would be

8 $$ \begin{array}{l}I=F{K}_C\left\{{\left({\left[N{a}^{+}\right]}_0 \exp \left(-\frac{FV}{2RT}\right)\right)}^{V_{Na}}\cdot {\left({\left[HC{O_3}^{-}\right]}_0 \exp \left(\frac{FV}{2RT}\right)\right)}^{v_{HC{O}_3}}-{\left({\left[N{a}^{+}\right]}_i \exp \left(\frac{FV}{2RT}\right)\right)}^{V_{Na}}\cdot {\left({\left[HC{O_3}^{-}\right]}_i \exp \left(-\frac{FV}{2RT}\right)\right)}^{v_{HC{O}_3}}\right\}\hfill \\ {}+{P}_s{Z}_s^2\frac{FV}{RT}\kern0.5em \frac{\left[S\right]i-{\left[S\right]}_0 \exp \left(-{Z}_s\frac{FV}{RT}\right)}{1- \exp \left(-{Z}_s\frac{FV}{RT}\right)}\hfill \end{array} $$

At V_I=0_ of the electrogenic NBC transporter plus one channel system

9 $$ \begin{array}{l}F{K}_c\left\{{\left({\left[N{a}^{+}\right]}_o \exp \left(-\frac{F{V}_{I=0}}{2RT}\right)\right)}^{\nu_{Na}}\cdot {\left({\left[HC{O_3}^{-}\right]}_o \exp \left(\frac{F{V}_{I=0}}{2RT}\right)\right)}^{\nu_{HC{O}_3}}\right.\\ {}\left.-{\left({\left[N{a}^{+}\right]}_i \exp \left(\frac{F{V}_{I=0}}{2RT}\right)\right)}^{\nu_{Na}}\times {\left({\left[HC{O_3}^{-}\right]}_i \exp \left(-\frac{F{V}_{I=0}}{2RT}\right)\right)}^{\nu_{HC{O}_3}}\right\}+{P}_s \exp \left(\frac{F^2{V}_{I=0}}{RT}\right)\frac{{\left[{s}^{+}\right]}_i-{\left[{s}^{+}\right]}_o \exp \left(-{z}_s\frac{F{V}_{I=0}}{RT}\right)}{1- \exp \left(-{z}_s\frac{F{V}_{I=0}}{RT}\right)}=0\end{array} $$

We can see that even with one additional channel, this equation contains more than one unknown such as K_c_, P_s_ and ν_HCO3_. What we measure in the electrophysiological experiments is V_I=0_. V_I=0_ is a complicated non-additive function of E_NBC_. A simple expression for the relationship between stoichiometry and reversal potential is not obtained. We will see a similar situation when there is one additional electrogenic cotransporter transporting ions s1 and s2 with involved function K_a_, valence Z_s1_ and Z_s2_, stoichiometry ν_s1_ and ν_s2_ respectively:

10 $$ \begin{array}{l}{I}_M=F{K}_c\left\{{\left({\left[N{a}^{+}\right]}_o \exp \left(-\frac{FV}{2RT}\right)\right)}^{\nu_{Na}}\cdot {\left({\left[HC{O_3}^{-}\right]}_o \exp \left(\frac{FV}{2RT}\right)\right)}^{\nu_{HC{O}_3}}\right.\left.-{\left({\left[N{a}^{+}\right]}_i \exp \left(\frac{FV}{2RT}\right)\right)}^{\nu_{Na}}\cdot {\left({\left[HC{O_3}^{-}\right]}_i \exp \left(-\frac{FV}{2RT}\right)\right)}^{\nu_{HC{O}_3}}\right\}\\ {}+F{K}_a\left\{{\left({\left[s1\right]}_o \exp \left(-\frac{FV{z}_{s1}}{2RT}\right)\right)}^{\nu_{s1}}\cdot {\left({\left[s2\right]}_o \exp \left(-\frac{FV{z}_{s2}}{2RT}\right)\right)}^{\nu_{s2}}\right.\left.-{\left({\left[s1\right]}_i \exp \left(\frac{FV{z}_{s1}}{2RT}\right)\right)}^{\nu_{s1}}\cdot {\left({\left[s2\right]}_i \exp \left(\frac{FV{z}_{s2}}{2RT}\right)\right)}^{\nu_{s2}}\right\}\end{array} $$

Again, a simple expression for the relationship between stoichiometry and reversal potential is not obtained.

**Figure 6:**
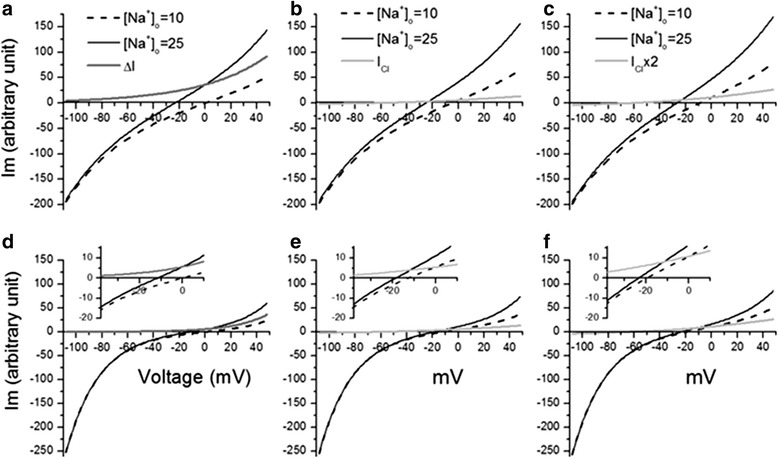
**Computational simulation of membrane currents and reversal potentials.** Addition of a Cl^−^ conductance (G_Cl_) has a significant impact on ΔE_rev_ and therefore biases the estimation of q of NBC. Based on Eq. 2, currents were calculated with the same conditions as our whole-cell patch-clamp experiments for estimation of q of NBCe2-C and NBCe1-A: [HCO_3_^−^]_i_ = [HCO_3_^−^]_o_ = 25, [Na^+^]_i_ =10 mM assuming q = 2 **(panels a**, **b and c)** or q = 3 **(panels d, e and f)**. **a)** I-V curves when bath solution switched from [Na^+^]_o_ =10 mM to 25 mM and the delta current (ΔI, the dark gray line). **b**) I-V curves when a relatively small G_Cl_ was present (light gray line) and the bath solution switched from [Na^+^]_o_ =10 mM to 25 mM. **C)** I-V curves when a relatively larger Cl^−^ conductance (2 x G_Cl_) was present (light gray line) and with the same bath solution switch as **b)**. **(d)**, **(e)** and **(f)** show the same operations as **(a)**, **(b)** and **(c)** respectively except assuming q = 3. The insets in panel **(d)**, **(e)** and **(f)** illustrate V_I=0_ by enlarging the local areas around I = 0. Y-axis’s are membrane currents of arbitrary unit for comparison purposes.

We performed a computational simulation of membrane currents and reversal potentials to show how a conductance in addition to electrogenic NBC transport affects the measurement of V_I=0_ and thus the estimate of q for this electrogenic NBC. Based on Eq. 2, currents were calculated with the same conditions as our whole-cell patch-clamp experiments for estimating q (delta current method above) of NBCe2-C: [HCO_3_^−^]_i_ = [HCO_3_^−^]_o_ = 25, [Na^+^]_i_ =10mM. Assuming q = 2, Figure 6a shows I-V curves and V_I=0_s when the bath solution switched from [Na^+^]_o_ =10 mM to 25 mM and the delta current (ΔI). The stoichiometry ratios estimated either with the ΔE_rev_ or ΔI methods are equivalent when there was no conductance other than the electrogenic NBC transporter (Table 2). However, if a small Cl^−^ conductance (compared to the conductance of the NBC-mediated current) was present, simulation with Eq. 8 showed that both V_I=0_ values at [Na^+^]_o_ = 10 mM and [Na^+^]_o_ = 25 mM shifted toward more negative value, but the shifts for the two conditions were different (Figure 6b). Therefore ΔE_rev_ differed from that obtained without the Cl^−^ conductance and leads to a different estimate of q = 2.17. When the Cl^−^ conductance was doubled, the estimate of q became 2.33 (Figure 6c). When we input q = 3 in the simulation, the estimate was 3 in the absence of any other conductance. After introducing either a small Cl^−^ conductance G_Cl_ or 2 x G_Cl_ (same as above), the estimate of q became 4.96 and 7.2 respectively with the ΔE_rev_ method (Figure 6d,e and f; note the insets; Table 2). However as shown in Table 2, the value of q determined using the ΔI method was unaffected by addition of a G_Cl_ on the membrane. Specifically, the ΔI-V curves in the absence, presence of small or large G_Cl_ were identical. Therefore, the currents mediated by other channels had been eliminated in the procedure and had no effect on the estimation of q.

**Table 2:** **Computational simulation of ΔI and ΔE**_**rev**_ **methods to estimate q in the absence or presence of a Cl** ^**−**^ **channel**

**[HCO**_**3**_^**−**^ **]**_**i**_ **= [HCO**_**3**_^**−**^ **]**_**o**_ **= 25**	**V**_**I=0**_ **(mV)**		**ΔE**_**rev**_ **(mV)**	**q (ΔE**_**rev**_ **)**	**ΔI**_**2**_ **/ ΔI**_**0**_ **(V**_**2**_ **= 12 mV)**	**q (ΔI)**
**[Na** ^**+**^ **]**_**i**_ **=10 mM**	**[Na** ^**+**^ **]**_**o**_ **= 10**	**[Na** ^**+**^ **]**_**o**_ **= 25**				
q = 2	0	−23.5	−23.5	2.0	1.263	2.0
q = 3	0	−11.75	−11.75	3.0	1.595	3.0
q = 2, + G_Cl_	−5.1	−25.3	−20.2	2.17	1.263	2.0
q = 3, + G_Cl_	−12.8	−18.75	−5.95	4.96	1.595	3.0
q = 2, + 2 x G_Cl_	−9.2	−26.9	−17.7	2.33	1.263	2.0
q = 3, +2 x G_Cl_	−19.7	−23.5	−3.8	7.2	1.595	3.0

G_Cl_ represents a Cl^−^ conductance in the conditions of [Cl^−^]_i_ = 12 and [Cl^−^]_o_ =125 mM. Column q (ΔE_rev_) represent q values estimated with ΔE_rev_ method. Column q (ΔI) represent q values estimated with ΔI method.

We then simulated NBCe1-A transport in conditions similar to the proximal tubule cells in the rat kidney where the ionic concentrations (in mM) were [HCO_3_^−^]_o_ = 24, [HCO_3_^−^]_i_ = 13.4, [Na^+^]_o_ = 150 and [Na^+^]_i_ =17 mM [26]. In addition to NBCe1-A, the Na^+^/D-glucose cotransporter SGLT2 was modeled in the simulation. SGLT2 is expressed in the apical membrane of proximal tubule cells and exhibits a transport stoichiometry of 1 Na^+^: 1 glucose [27]. One positive charge moves across the membrane per transport cycle. An extracellular glucose concentration [G]_o_ = 5 mM and intracellular [G]_i_ = 1 mM were substituted into Eq. 10 assuming q = 2 or 3 for NBCe1-A. Table 3 shows the V_I=0_ values when [Na^+^]_o_ = 150 and when [Na^+^]_o_ was switched to 100 in the absence and presence of SGLT2. The simulation also provided estimated q values by ΔE_rev_ and ΔI methods. The stoichiometry ratios estimated either with the ΔE_rev_ or ΔI methods were equivalent when SGLT2 was absent. However, when SGLT2 was present, q was 2.55 estimated with the ΔE_rev_ method when the actual value in the simulation was 3 (Table 3). The presence of SGLT2 prevents any definitive determination as to whether the stoichiometry of NBCe1-A is q = 2 or q = 3.

**Table 3:** **Simulation of ΔI and ΔE**_**rev**_ **methods to estimate q in conditions similar to rat proximal tubule in the absence or presence of a Na** ^**+**^ **/D-glucose cotransporter**

**[HCO**_**3**_^**−**^ **]**_**o**_ **= 24, [HCO**_**3**_^**−**^ **]**_**i**_ **= 13.4**	**V**_**I=0**_ **(mV)**		**ΔE**_**rev**_ **(mV)**	**q (ΔE**_**rev**_ **)**	**ΔI**_**2**_ **/ΔI**_**1**_ **V**_**2**_ **-V**_**1**_ **= 10 mV**	**q (ΔI)**
**[Na** ^**+**^ **]**_**i**_ **= 17 mM**	**[Na** ^**+**^ **]**_**o**_ **= 150**	**[Na** ^**+**^ **]**_**o**_ **= 100**				
q = 2	−89.6	−78.75	10.85	2.0	1.205	2.0
q = 3	−52.6	−47.2	5.4	3.0	1.45	3.0
q = 2, + Glu	−73.6	−62.75	10.85	2.0	1.205	2.0
q = 3, + Glu	3.2	10.2	7.0	2.55	1.45	3.0

Glu represent a Na^+^/D-glucose cotransporter SGLT2 in the conditions of glucose concentrations [G]_o_ = 5, [G]_i_ = 1 and [Na^+^]_o_ = 150, [Na^+^]_i_ = 17 (in mM). Column q (ΔE_rev_) represent q values estimated with ΔE_rev_ method. Column q (ΔI) represent q values estimated with ΔI method.

These results indicate that the ΔE_rev_ method can significantly bias the estimate depending on the magnitude and electrophysiological properties (e.g. the I-V relationship) of other channels and electrogenic transporters if there are any, while the ΔI method gives a more accurate estimate of the transport stoichiometry q.

## Discussion

In this study, we have demonstrated the development and utility of a new method for estimating the transport stoichiometry of electrogenic transport proteins. With this ΔI method, one subtracts the currents due to channels and transporters other than the one under study and thereby obtains the stoichiometry of the transporter without the need for a specific inhibitor. Using this method, we showed that the transport stoichiometry of the bicarbonate cotransporter NBCe2-C expressed in HEK-293 cells is 2 HCO_3_^−^: 1 Na^+^ that is consistent with the results obtained using the conventional reversal potential method with the inhibitor DIDS. A transport stoichiometry ratio of 2 was also obtained for NBCe1-A with the ΔI method that is consistent with the data obtained previously using the conventional reversal potential method with DIDS [25]. In addition, we demonstrated that, with computational simulation, the estimation of q obtained using the new ΔI method was equivalent to that obtained with the conventional ΔE_rev_ methods when an electrogenic NBC transporter was the only transport mechanism in the cell membrane. However, if a chloride channel or a glucose cotransporter SGLT2 was present in the membrane, our simulations showed that the ΔE_rev_ method significantly biased the estimate of the transport stoichiometry q, while the ΔI method gave accurate results.

The method proposed in this study is based on Eq. 2 from Heinz [23] that describes the functional relationship between flux of a transporter and the concentrations of transport ions/substrates and the membrane voltage [24]. Unlike the GHK formulation that assumes independence of ion movement across the membrane [13] and does not involve the concept of stoichiometry, Eq. 2 explicitly expresses coupling of Na^+^ and HCO_3_^−^ (both are voltage dependent) as a product and the stoichiometry as a power of the concentrations and voltage. Linearity of the current and voltage relation is not a presumption for Eq. 2 nor is it for the GHK equations [17,18]. Non-linearity of the I-V curves results from: 1) the GHK equation is based on solubility-diffusion theory. In GHK current equation, the current is an exponential function of the voltage. Similarly Eq. 2 shows that flux is an exponential function of voltage; 2) transport mechanisms of membrane channels or transporters represented by the permeability term Ps in GHK equations and K_c_ in Eq. 2 may be voltage dependent. With the conventional E_rev_ method, if the transporter under study is the only electrogenic pathway, this non-linearity would not be a problem since the current is 0 and at this point, the voltage is the reversal potential under the conditions of the experimental substrate concentrations. However, if there are other channels or electrogenic transporters in the membrane and if a specific inhibitor is not available, V_I=0_ that can be measured is not the reversal potential for the transporter under study, but rather is the voltage at a point on the I-V curve where the net result of the transporter current under study and currents mediated by other transporters and channels is 0. The alternative ΔE_rev_ method is problematic in that the assumption of reversal potential additivity is inconsistent with non-linearity property of GHK equations and Eq. 2. This is solved by employing the ΔI method where the contribution of other channels or transporters can be eliminated without the assumption of E_ver_ additivity.

If we assume that an electrogenic NBC transporter has a fixed transport stoichiometry, if the only ions that cross the cell membrane are Na^+^ and HCO_3_^−^ , from Eq. 2 we have

11 $$ I=F{K}_C\left\{{\left({\left[N{a}^{+}\right]}_0 \exp \left(-\frac{FV}{2RT}\right)\right)}^{V_{Na}}\cdot {\left({\left[HC{O_3}^{-}\right]}_0 \exp \left(\frac{FV}{2RT}\right)\right)}^{v_{HC{O}_3}}-{\left({\left[N{a}^{+}\right]}_i \exp \left(\frac{FV}{2RT}\right)\right)}^{V_{Na}}\cdot {\left({\left[HC{O_3}^{-}\right]}_i \exp \left(-\frac{FV}{2RT}\right)\right)}^{v_{HC{O}_3}}\right\} $$

When I = 0, we have

12 $$ {\left({\left[N{a}^{+}\right]}_o\right)}^{\nu_{Na}}{\left({\left[HC{O_3}^{-}\right]}_o\right)}^{\nu_{HC{O}_3}} \exp \left(\left({\nu}_{HC{O}_3}-{\nu}_{Na}\right)\frac{F{V}_{I=0}}{2RT}\right)={\left({\left[N{a}^{+}\right]}_i\right)}^{\nu_{Na}}{\left({\left[HC{O_3}^{-}\right]}_i\right)}^{\nu_{HC{O}_3}} \exp \left(\left({\nu}_{Na}-{\nu}_{HC{O}_3}\right)\frac{F{V}_{I=0}}{2RT}\right) $$

13 $$ \begin{array}{l} \ln \left({\left({\left[N{a}^{+}\right]}_o\right)}^{\nu_{Na}}{\left({\left[HC{O_3}^{-}\right]}_o\right)}^{\nu_{HC{O}_3}}\right)+\left({\nu}_{HC{O}_3}-{\nu}_{Na}\right)\frac{F{V}_{I=0}}{2RT}\\ {}= \ln \left({\left({\left[N{a}^{+}\right]}_i\right)}^{\nu_{Na}}{\left({\left[HC{O_3}^{-}\right]}_i\right)}^{\nu_{HC{O}_3}}\right)+\left({\nu}_{Na}-{\nu}_{HCO3}\right)\frac{F{V}_{I=0}}{2RT}\end{array} $$

Therefore,

14 $$ {V}_{I=0}=\frac{RT}{F\left({\nu}_{HC{O}_3}-{\nu}_{Na}\right)} \ln \left(\frac{{\left({\left[N{a}^{+}\right]}_i\right)}^{\nu_{Na}}{\left({\left[HC{O_3}^{-}\right]}_i\right)}^{\nu_{HC{O}_3}}}{{\left({\left[N{a}^{+}\right]}_o\right)}^{\nu_{Na}}{\left({\left[HC{O_3}^{-}\right]}_o\right)}^{\nu_{HC{O}_3}}}\right)={E}_{NBC} $$

This is essentially Eq. 1 if we take ν_Na_ = 1 and q = ν_HCO3_/ν_Na_. Starting from here, the widely used delta reversal potential method to estimate stoichiometry [2,15,16] can be easily derived:

When we change Na^+^ concentration in the bath solution from [Na^+^]_o1_ to [Na^+^]_o2_, we have

15 $$ V{2}_{I=0}=\frac{RT}{F\left(q-1\right)} \ln \frac{{\left[N{a}^{+}\right]}_i{\left({\left[HC{O_3}^{-}\right]}_i\right)}^q}{{\left[N{a}^{+}\right]}_{o2}{\left({\left[HC{O_3}^{-}\right]}_o\right)}^q} $$

Then, delta reversal potential ΔE_rev_ would be

16 $$ \begin{array}{l}\varDelta {E}_{rev}=V{2}_{I=0}-V{1}_{I=o}=\frac{RT}{F\left(q-1\right)}\left\{ \ln \frac{{\left[N{a}^{+}\right]}_i{\left({\left[HC{O_3}^{-}\right]}_i\right)}^q}{{\left[N{a}^{+}\right]}_{o2}{\left({\left[HC{O_3}^{-}\right]}_o\right)}^q}- \ln \frac{{\left[N{a}^{+}\right]}_i{\left({\left[HC{O_3}^{-}\right]}_i\right)}^q}{{\left[N{a}^{+}\right]}_{o1}{\left({\left[HC{O_3}^{-}\right]}_o\right)}^q}\right\}\\ {}=\frac{RT}{F\left(q-1\right)} \ln \frac{{\left[N{a}^{+}\right]}_{o1}}{{\left[N{a}^{+}\right]}_{o2}}\end{array} $$

From the above operations, we can see that reversal potential method, the ΔE_rev_ method and the ΔI method to estimate transport stoichiometry all have the same theoretical foundation (such as Eq. 2 and same assumptions). Moreover, they are equivalent if the electrogenic transporter under investigation is the only conductive process in the membrane.

However, if there are endogenous channels and electrogenic transporters other than the one under study, the relationship of ion activities and transport stoichiometry and reversal potential becomes very complicated as we can see in Eq. 8, Eq. 9 and Eq. 10. Therefore, a method to eliminate the confounding effects of additional transporters and channels on reversal potentials by simple subtraction of V_I=0_ is not valid. Our simulation results also indicate that the commonly used ΔE_rev_ method in this instance would not be accurate. The error increases as the currents mediated by other transporters and channels increase (Table 2) relative to the transporter under investigation.

Transport parameters of an electrogenic secondary active transport like K_c_ are affected by many factors. How a given transport process responds theoretically to an electro-chemical gradient depends on the type of the transport kinetic models utilized, e.g. “affinity model”, “velocity model” or “mixed model” as described by Heinz [24], and whether the loaded or the unloaded carrier bears an electrical charge. Heinz [23] originally introduced equation 2 and referred to K_c_ as a function of mobility and concentrations of the free and loaded carrier, respectively, and hence may vary with the degree of saturation. In our approach, we made two assumptions that are implicitly shared with the ΔE_rev_ method: 1) K_c_ is constant in certain voltage range and does not vary when the concentration of the substrate of choice in the study ([Na^+^]_o_ in this study) changes; 2) the sum of currents ${\displaystyle \sum_j{I}_j}$ mediated by other channels and transporters in the membrane as a function of V does not change when the substrate concentration is altered [2,15,16]. Based on these two assumptions the two methods offer benefits such as experimentally straightforward as changing the concentrations of a substrate without the need for specific blockers and share similar limitations. The difference between ΔI and ΔE_rev_ method in terms of assumption 2 is that with the ΔI method, ${\displaystyle \sum_j{I}_j}$ can be completely eliminated (Eq. 4) if it does not change when the substrate ([Na^+^]_o_ in this study) is altered. On the contrary, with the ΔE_rev_ method, as long as ${\displaystyle \sum_j{I}_j}$ is not negligible, the confounding effects of ${\displaystyle \sum_j{I}_j}$ on V_I=0_ can not be eliminated and biases the estimation of q as shown in Figure 6 and Table 2 and Table 3, even if it does not change when the substrate concentration varies.

In practice, ways to circumvent the limitations due to the above assumptions include: 1) using a smaller concentration change of the substrate, as long as it induces a significant delta current; 2) changing the concentrations of a particular substrate with less possibility of involving other electrogenic transporters. For example, in the case of electrogenic Na^+^-coupled glucose or amino acid transporters, one would choose to change either glucose or amino acids respectively rather than Na^+^.

In this study, we changed [Na^+^]_o_ from 10 to 25 mM because: 1) HCO_3_^−^ partakes in a volatile buffer system that involves pCO_2_ to keep the pH constant. pH would be stable when [HCO_3_^−^]_o_ is unaltered; 2) switching [Na^+^]_o_ from 10 to 25 mM would induce a significant delta current [15] and 3) at these relatively low concentrations, the possibility of transport saturation would be small, therefore variation of K_c_ in Eq. 2 and Eq. 3 would be minimized. We assigned V_1_ = 0 in the above application, therefore in the conditions of ${\left[N{a}^{+}\right]}_i={\left[N{a}^{+}\right]}_o and\ {\left[HC{O_3}^{-}\right]}_i={\left[HC{O_3}^{-}\right]}_o,{I}_M={\displaystyle \sum_j{I}_j}$ is well defined and it is not close to 0. In addition, we assigned a V_2_ that is not far from 0 (+12 mV in this study), thus possible variation of K_c_ under extreme voltages can be minimized.

More detailed kinetic descriptions of the transport rate in order to characterize the entire I-V relationship rely on a detailed understanding of the molecular transport steps [28-30]. This is not necessary for the purposes of our formulation, because we implicitly analyze the portion of the I-V relationship that is close to the E_rev_ i.e., V_1_ = 0 when [Na^+^]_i_ = [Na^+^]_o_ and [HCO_3_^−^]_i_ = [HCO_3_^−^]_o_.

The accuracy of stoichiometry estimation using whole-cell patch-clamp recordings also depends on the accuracy of whole-cell current measurement and the voltages applied to the cell membrane from the patch-clamp amplifier. The drift of the junction potential between the patch pipette solution and the Ag/AgCl coated wire that connects to the headstage of the amplifier is a major source of unstable current recording especially when the Cl^−^ concentration in the pipette is low [22]. We used a micro-agar salt bridge of 2 M KCl in the patch pipette that minimized the junction potential drift and therefore stabilized the whole-cell current measurements [22].

## Conclusions

We developed a new delta current (ΔI) method for estimating transport stoichiometry of electrogenic transporters based on a simplified model for electrogenic secondary active transport by Heinz (1981). We showed that this model reduces to the conventional reversal potential method when the transporter under study is the only electrogenic transport on the membrane. When there are other electrogenic transport processes such as ion channels or transporters, the ΔI method eliminates their contribution in estimation of q. We tested this new ΔI methodology in HEK-293 cells expressing the electrogenic SLC4 sodium bicarbonate cotransporters NBCe2-C and NBCe1-A, as well as using computational simulations. Our simulations demonstrated that the ΔE_rev_ method introduces significant error when other channels or electrogenic transporters are present on the membrane with a significant conductance relative to the transporter under study, and that the ΔI equation accurately calculates the stoichiometric ratio. Our new ΔI method can be readily extended to the analysis of other electrogenic transporters.

## Abbreviations

CMV: Cytomegalovirus
DIDS: 4,4′-Diisothiocyanatostilbene-2,2′-disulfonic acid
EGFP: Enhanced Green Fluorescent Protein
Eq: Equation
E_rev_: Reversal potential
F: Faraday’s constant
GHK: Goldman-Hodgkin-Katz
HEPES: 4-(2-Hydroxyethyl)-1-piperazineethanesulfonic acid
I-V: Current-voltage
NBC: Sodium bicarbonate cotransporter
R: Gas constant
SGLT2: Sodium-coupled glucose transporter 2
T: Absolute temperature
ΔE_rev_: Delta reversal potential
ΔI: Delta current
